# Delayed Appearance of a Traumatic Fetal Intracranial Hemorrhage

**DOI:** 10.1155/2018/1465034

**Published:** 2018-02-27

**Authors:** Kelly Yamasato, Nicole Kurata, Dena Towner

**Affiliations:** Department of Obstetrics, Gynecology, and Women's Health, University of Hawaii John A Burns School of Medicine, Honolulu, HI, USA

## Abstract

**Background:**

Fetal intracranial injury is a potentially devastating sequelae of maternal trauma, but there is little guidance regarding fetal evaluation in this setting.

**Case:**

A 23-year-old woman at 27-week gestation was admitted after a high-speed motor vehicle accident. The initial obstetrical ultrasound was unremarkable, but persistently minimal fetal heart rate variability was observed. Ultrasound on day 3 after the accident showed an intracranial hyperechogenic lesion and subdural fluid collection. The neonate, following an uneventful birth at 39 weeks, had seizures and abnormal muscle tone. MRI was consistent with in utero intracranial hemorrhage.

**Conclusion:**

Serial fetal imaging following maternal trauma, particularly when accompanied by abnormal fetal heart rate tracings, should be considered when fetal injury is a concern, even in the setting of a normal initial ultrasound.

## 1. Introduction

Physical trauma is common in pregnancy, affecting 1 in 12 pregnant women [1]. Fetal intracranial hemorrhage, often associated with a poor prognosis [2, 3], is a rare but potentially devastating consequence of maternal trauma. The literature on fetal intracranial injury secondary to maternal trauma is limited to case reports and case series. While an obstetrical ultrasound is suggested for maternal trauma that warrants extended fetal surveillance [1], there is little guidance on the timing and follow-up for these ultrasounds. We report a case of maternal trauma with a delayed presentation of fetal intracranial injury.

## 2. Case

**Figure 1:**
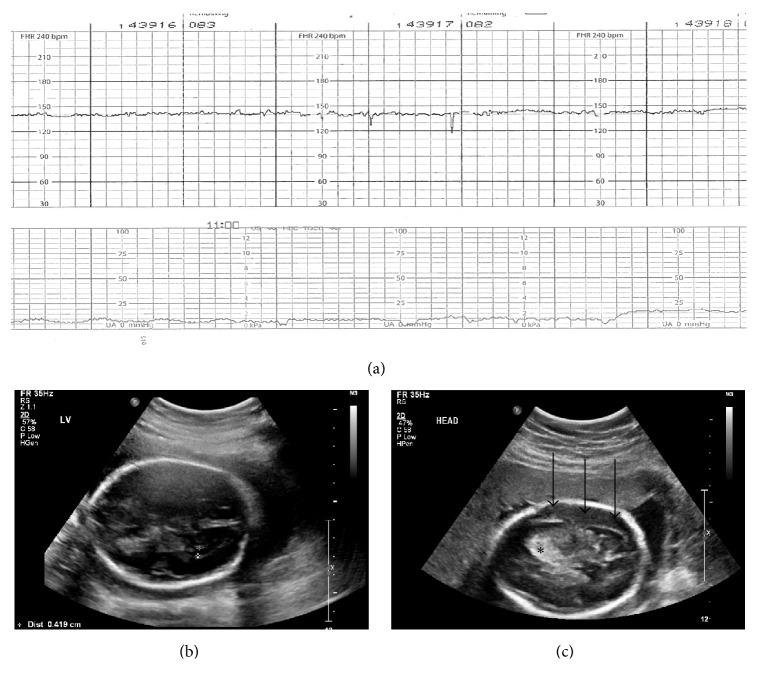
Fetal heart rate and evolving ultrasound findings following fetal intracranial injury. (a) Fetal heart rate showed a normal baseline with persistent minimal variability and no accelerations or decelerations (b) 34 hours after trauma: fetal intracranial anatomy largely unremarkable as demonstrated by this transverse view at the level of the lateral ventricle. (c) 62 hours after trauma: a large hyperechoic lesion in the frontal lobe is visualized (*∗*) as well as findings consistent with a subdural hematoma (arrows).

A 23-year-old gravida 2 para 1 woman at 27-week gestation was admitted following a high-speed motor vehicle accident. She was a restrained front seat passenger in a car that rear-ended a parked vehicle at 70 miles per hour. Injuries included a left hip dislocation and multiple closed pelvic fractures involving the bilateral hips and the bilateral pubic rami. She was alert and normotensive on admission. Fetal heart tones were noted to have a baseline of 140 beats per minute with minimal variability and no accelerations or decelerations (Figure 1(a)). The fetus was in footling breech presentation.

She received a course of betamethasone. An ultrasound done approximately 34 hours into admission showed unremarkable intracranial anatomy (Figure 1(b)) with an anterior placenta and no evidence of abruption. The biparietal diameter (BPD) was 71 mm (81%ile) and head circumference (HC) 263 mm (65%ile). The amniotic fluid volume was normal at 12.3 cm.

In the subsequent days of admission the fetal heart tones demonstrated persistent minimal variability without accelerations or decelerations. The patient was receiving fentanyl and it was speculated that narcotics were contributing to the decreased variability. Approximately 62 hours into admission a biophysical profile (BPP) was performed. The BPP returned 6 of 8 (off 2 points for absent breathing). However at this time a hyperechoic lesion in the left frontal cortex was noted, extending to the left lateral ventricle with an additional fluid collection concerning for a left subdural hematoma (Figure 1(c)). The middle cerebral artery Doppler peak systolic velocity was normal at 1.04 MoM. With the knowledge of a central nervous system (CNS) insult and the absence of fetal anemia, the abnormal fetal heart rate pattern was assessed to be a manifestation of the injury rather than placental dysfunction or fetal acidosis, thereby avoiding an unnecessary preterm delivery. By hospital day 6 the fetal heart tones became reactive and she was discharged home.

**Figure 2:**
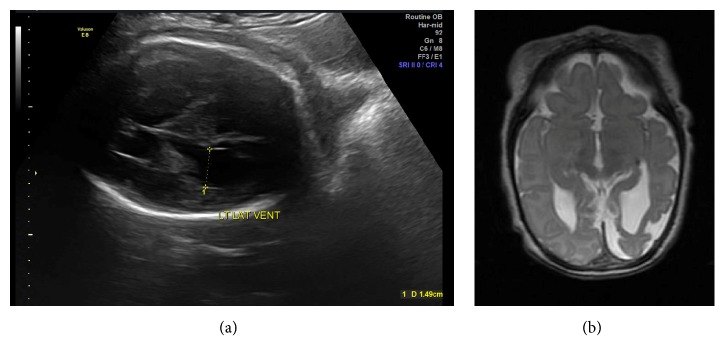
Fetal intracranial imaging and postnatal follow-up. (a) Ultrasound at 35-week gestation demonstrates unilateral ventriculomegaly. (b) MRI at day 4 of life shows cystic encephalomalacia of the bilateral frontal and left parietal lobes and artifact consistent with a previous intraventricular hemorrhage, hemorrhagic contusion, and subdural hematoma.

She presented for follow-up ultrasound at 30-week gestation. A left subdural hypoechogenic lesion and mild left ventriculomegaly were present. In addition, there was no interval head growth (BPD 67 mm, HC 269 mm). Follow-up ultrasound at 35 weeks again showed mild left ventriculomegaly (Figure 2(a)) and minimal head growth (BPD 72 mm, HC 273 mm).

Delivery was by an uncomplicated scheduled repeat cesarean delivery at 39 weeks gestation. APGARs were 8 and 8 at 1 and 5 minutes, respectively, with umbilical artery cord pH of 7.16 and base excess of −4.3. The neonate required continuous positive airway pressure in the delivery room. Birthweight was 3033 gm, but severe microcephaly was present with a head circumference of 31.8 cm (<3%ile). Neurologic findings also included seizure-like activity, lower extremity hyperreflexia, and increased truncal tone. Brain MRI on day of life 4 was consistent with cystic encephalomalacia of the bilateral frontal lobes and left parietal lobe. There was also artifact suggestive of hemosiderin deposits, consistent with prior left intraventricular hemorrhage, hemorrhagic contusion, and subdural hematoma, but no indications of skull fracture (Figure 2(b)). There was no coagulopathy. At 8 months of age the infant is undergoing care for cerebral palsy with quadriplegic involvement.

## 3. Discussion

While fetal intracranial hemorrhage is a known potential sequelae of maternal trauma, this is among the first reported cases of the delayed appearance of such hemorrhage. The events of this case demonstrate the possibility of severe fetal intracranial injury even in the setting of unremarkable initial imaging. It also helps delineate the timing of imaging as the CNS findings at 34 hours after injury were not evident enough to be detected on a thorough fetal exam, but by 62 hours the findings were striking enough to be noted at the time of a BPP, which is not focused on the CNS. The exact timing of the intracranial bleeding cannot be determined with certainty. However, given the extent of the blunt forces and maternal pelvic injuries, as well as the decreased fetal heart rate variability upon admission, it is suspected that the fetal injury occurred at the time of the trauma with a delayed presentation. In addition, there were no subsequent maternal hypoxic or hypotensive events that would account for a later onset of fetal injury.

There are multiple potential underlying reasons for a delayed presentation. Certainly, more subtle findings of intracranial hemorrhage could have been present on the initial ultrasound and went undetected, and on retrospective review there was a hint of increased echogenicity on the first set of images. However the extent of the abnormality was significantly more pronounced on the second ultrasound. A small but ongoing bleed could also have been present, though a significant hemorrhage is unlikely in the setting of a normal middle cerebral artery Doppler. It is also well known that the appearance of an intracranial hemorrhage evolves with the formation and then breakdown of the hematoma. The initial intracranial hemorrhage is liquid with highly oxygen-saturated hemoglobin. Within hours a heterogeneous clot forms and subsequently retracts. Serum is expelled from the clot into the surrounding tissue, which also undergoes vasogenic edema. As the injury evolves, red blood cell lysis and hematoma resolution occur with hemosiderin deposition. The resolved hematoma may present as a cystic or collapsed brain defect [4]. Ultrasound findings in intracranial hemorrhage may therefore include hyperechogenic lesions consistent with blood clot with subsequent ventriculomegaly, hydrocephalus, and parenchymal atrophy [2, 3], as was consistent with this case. The arrest in head growth in this case is less commonly reported in the setting of fetal intracranial hemorrhage but has been associated with fetal head trauma. The normal fetal head size on admission also suggests injury at the time of the trauma to be the underlying etiology.

Strigini et al. described two cases of fetal intracranial hemorrhage following minor maternal trauma in which an ultrasound within 24 hours of the event appeared normal, but in which injury was visualized several weeks later [5]. However, the minor nature of the trauma in both of these cases and the extended time interval between the trauma and abnormal ultrasound findings lead to the consideration for a different pathophysiology.

Fetal intracranial hemorrhage is identified in up to 1 per 1000 births in referral centers. Intraventricular hemorrhage is the most common location of bleeding in the fetus [2, 3], but with parenchymal or subdural/subarachnoid involvement severe neurologic impairment or perinatal mortality occurs in up to 90% of cases [2]. A systematic review of fetal subdural or epidural hematoma after maternal trauma identified 14 cases, 9 of them secondary to motor vehicle accidents as with our patient. Of these 14 cases 3 patients experienced intrauterine fetal demise and 3 neonatal demise [6]. While true mortality and morbidity rates are unknown, the seemingly high risks for these outcomes speak to the importance of timely diagnosis. In addition to maternal trauma, other precipitating events associated with fetal intracranial hemorrhage include in utero asphyxia, fetal coagulopathy, and infection. The largest review to-date identified a history of maternal trauma in 10 of 109 cases of fetal intracranial hemorrhage [7].

The literature on fetal intracranial hemorrhage secondary to maternal trauma is limited to case reports and case series. A theorized mechanism of fetal head injury with blunt maternal trauma is fetal skull compression between the pubic symphysis and sacrum [8]. Severe fetal head injury has also been reported in the setting of airbag deployment [8–10]. In this case, the fetus was breech on admission, and breech presentation at the time of the accident likely precludes skull compression in the pelvis as the underlying mechanism of injury. Even if cephalic, the small head size of a 27-week fetus in the absence of a displaced pelvic fracture makes skull compression unlikely. In addition, there was no airbag deployment. However, the patient was wearing a seat belt, and the fetal head could have conceivably been compressed between the belt and maternal spine given the rapid deceleration from 70 mph.

An obstetrical ultrasound has been recommended for all pregnant trauma patients with a viable pregnancy who are admitted for greater than 4 hours for fetal monitoring [1]. Given the findings of this case, we suggest consideration for a repeat ultrasound at least 72 hours after trauma if there are other risk factors for fetal injury such as significant maternal pelvic trauma, decreased fetal heart rate variability for gestational age, or maternal hypoxia/hypotension. Ultrasound is the first-line evaluation in cases of fetal intracranial hemorrhage, while fetal MRI has also been suggested as an adjunct to ultrasound to evaluate the extent of involvement [2, 3]. A computed tomography scan, when obtained for maternal evaluation, has also been reported to demonstrate fetal intracranial hemorrhage [11], though this is not a preferred method of fetal evaluation.

Abnormal fetal heart rate tracings alone in the setting of maternal trauma are poor predictors of adverse obstetrical outcomes [1]. However, fetal heart rate abnormalities, including decreased fetal heart rate variability and a sinusoidal pattern, have been associated with fetal intracranial hemorrhage [12–14]. In this case, the minimal fetal heart rate variability that persisted, even as narcotic use decreased, heightened the suspicion for neurologic injury and aided in its diagnosis.

Fetal intracranial injury is a rare but serious consequence of maternal trauma that may not always be apparent on initial presentation. Prenatal diagnosis of such injuries is important for optimizing delivery plans and location. Thus, serial fetal imaging should be considered following maternal trauma, particularly in the setting of an abnormal fetal heart rate tracing.
